# Hepatoprotective effect of flavonoid rich fraction of *Sesbania grandiflora*: Results of *In vivo*, *in vitro*, and molecular docking studies

**DOI:** 10.1016/j.jaim.2024.101036

**Published:** 2024-09-06

**Authors:** Anitha Kuttiappan, Santenna Chenchula, Murugesan Vanangamudi, Shvetank Bhatt, Radhika Chikatipalli, P Shaila Bhanu, Nagaraju Bandaru

**Affiliations:** a Department of Pharmacology, School of Pharmacy and Technology Management (SPTM), SVKM's Narsee Monjee Institute of Management Studies (NMIMS) Deemed-to-University, Shirpur 425405, Maharashtra, India; b Department of Pharmacology, All India Institute of Medical Sciences (AIIMS), Bhopal 462020, Madhya Pradesh, India; c Amity Institute of Pharmacy, Amity University Madhya Pradesh, Gwalior, Madhya Pradesh, 474005, India; d School of Health Sciences and Technology, Dr. Vishwanath Karad MIT World Peace University, Pune 411038, Maharashtra, India; e Sri Venkateswara College of Pharmacy, Chittoor 517120, Andhra Pradesh, India; f Sree Vidyanikethan College of Pharmacy, Sree Sainath Nagar, Tirupathi 517102, Andhra Pradesh, India; g Department of Pharmacology, School of Pharmaceutical Sciences (SOPS), Sandip University, Nasik 422213, Maharashtra, India

**Keywords:** *Sesbania grandiflora*, Flavonoids, HepG2 cells, MTT assay, Hepatotoxicity

## Abstract

**Background:**

Phytochemicals and their derivatives are promising target drugs for various ailments and have served as therapeutic agents for several decades. Using *in vivo* and *in vitro* models and molecular docking, this study investigated the pharmacological potential of a flavonoid-rich fraction of the ethanolic extract of *Sesbania grandiflora* (SG).

**Objectives:**

This research aimed to determine whether flavonoid-rich whole-plant extracts of SGs have any cytoprotective or *in vivo* hepatoprotective effects. Additionally, the study was intended to elucidate the molecular connections between the discovered flavonoid flavonols and PPARα target proteins linked to liver problems, for which an in silico molecular docking investigation was performed.

**Materials and methods:**

To separate the flavonoid components, the entire *Sesbania grandiflora* plant was first extracted using ethanol as a solvent by soxhlet extraction. The resulting ethanolic extract was then fractionated. The cytoprotective and hepatoprotective properties were evaluated via *in vitro* and *in vivo* experiments. SGOT, SGPT, triglyceride, bilirubin, and total protein levels were used to evaluate hepatotoxicity in animal models. *In vitro* studies on Hepatocellular Carcinoma G2 (HepG2) cell lines have examined their cytotoxic effects and antioxidant activity. The most promising flavonoid-flavanol compounds were identified by conducting molecular docking studies against PPARα target protein (PDB ID: 3VI8) using MOE software.

**Results:**

*In vivo*, the serum levels of SGOT, SGPT, total triglyceride and total bilirubin were measured in experimental animals treated with the flavonoid-rich ethanolic extract of SG. Significant reductions in the levels of these hepatic injury markers were observed, indicating the hepatoprotective potential of the extract. Elevated levels of liver biomarkers in the untreated group indicated liver injury or dysfunction. The treated groups showed significant restoration of these biomarkers, suggesting the hepatoprotective potential of *SG*. The IC_50_ value for the total flavonoid content of SG was 190.28 μg/ml, indicating its safety in inhibiting HepG2 cell growth. Flavonoid treatment decreased cell viability but did not affect antioxidant parameters in hepatocytes. In addition, SG restored the damaged hepatocyte architecture. Molecular docking studies revealed the binding affinities of flavonoids for PPARα. These findings suggest that a promising lead candidate for the development of therapeutic medicines against anti-TB drug-induced hepatotoxicity has been identified.

**Conclusion:**

Our findings demonstrate the hepatoprotective potential of the flavonoid-rich fraction of *Sesbania grandiflora* both *in vivo* and *in vitro*. This study provides valuable insights into its mechanism of action, highlighting its promising therapeutic application in the management of liver disorders. This study highlights the hepatoprotective and cytoprotective potential of the total flavonoid-rich fraction of SG.

## 1 Introduction

*Sesbania grandiflora (L.)* Pers, commonly known as “agathi,” is traditionally recognized for its potent medicinal properties and is frequently used to treat various diseases. Despite significant advancements in hepatology in recent years, hepatic disorders remain a global concern [1,2]^,^ as do other ailments, such as arthritis [3] and nociception [4,5]. Dietary foods rich in natural antioxidants have been proposed to prevent and treat liver damage. *Sesbania grandiflora*, a fast-growing tree belonging to the Fabaceae family, originated in India and Southeast Asia. Its regular and round leaves, large white flowers, and long, thin green beans characterize it. *Sesbania grandiflora* has various traditional properties, including antioxidant, cardioprotective, antiulcer [6], hepatoprotective [7], anti-inflammatory, anticancer [8], anxiolytic [9] and anticonvulsive activities [10]. Numerous plant-based products, such as *Glycyrrhiza glabra, Silybum marianum, Barleria montana*, and *Zingiber officinale* have been utilized clinically to treat a wide range of liver diseases. These herbal plants exert multiple effects on treating liver damage, primarily through their hepatoprotective and antioxidant properties [11].

Hepatic dysfunction involves cell necrosis, apoptosis, fibrosis, increased lipid peroxidation in tissues, and glutathione depletion [12]. Liver marker enzymes play a crucial role in assessing hepatic damage. Elevated levels of serum liver biomarkers indicate leakage of cellular contents and loss of physiological function of hepatocyte cell membranes [13]. HepG2 cell lines, which can maintain the differentiated parenchymal functions of normal liver cells and can be cultivated indefinitely, are widely used for *in vitro* studies [13]. *In vitro*, cultures utilizing human liver cells are considered highly effective and safe for toxicity and metabolic research [14]. The MTT (3-(4, 5-dimethylthiazolyl-2)-2, 5-diphenyltetrazolium bromide) assay, which employs a 96-well plate, was used in this study to assess cell proliferation, viability, and the biosafety of the plant extract in vitro. The findings of the present study revealed the hepatoprotective effects of an ethanolic extract of *Sesbania grandiflora.*

The evaluation of the hepatoprotective impact of the flavonoid-rich fraction of *Sesbania grandiflora* involves a multifaceted approach that includes *in vivo*, *in vitro*, and molecular docking investigations. This extensive inquiry is motivated by the urgent need to investigate the potential therapeutic effects of natural substances on liver function. With the increased global frequency of liver illnesses, there is an increasing demand for effective and safe alternative treatments. *Sesbania grandiflora,* recognized for its high flavonoid content, represents a prospective route for research due to its traditional use in a variety of therapeutic practices [15]. By undertaking *in vivo* investigations, researchers hope to gain significant insights into the physiological impact and potential medicinal applications of flavonoid fractions. In vitro investigations complement these findings by revealing the processes underlying the hepatoprotective effects of flavonoids and explaining their interactions at the cellular level [16]. Furthermore, molecular docking studies provide a computational perspective by revealing the binding affinity of flavonoids for specific liver enzymes or receptors involved in hepatoprotection. Collectively, these approaches seek to validate the hepatoprotective properties of the flavonoid-rich fraction of *Sesbania grandiflora*, providing a scientific foundation for further investigations of this plant as a natural therapy for liver disorders.

## 2 Materials and methods

### 2.1 Plant material

In the tirumala hills, chittoor district of andhra pradesh, India, the whole plant parts of *Sesbania grandiflora* were collected and validated by a botanist professor from Sri Venkateswara University in Tirupati named N. Yasodamma, along with specimen voucher number No: KL-20; 384 was collected by S Job Roger Binny for future use.

### 2.2 Extraction and fractionation of flavonoids

The whole plant of *Sesbania grandiflor*a was air-dried at room temperature and kibbled into a coarse powder, and 500 g of powdered material was subjected to soxhlet extraction for 72 h via a continuous hot percolation method using ethanol as a solvent [17,18]. The crude extract obtained under reduced pressure at 65 °C was evaporated by rotary evaporators, and the yield was 6.32%. The obtained ethanolic extract of *Sesbania grandiflora* (EESG) was subjected to fractionation, i.e., it was redissolved in 250 mL of distilled water and partitioned (2 × ) with an equal volume of *n*-butanol to obtain the flavonoid constituents [19]. The fractions were concentrated under reduced pressure and further subjected to *in vivo* and cytotoxicity studies.

### 2.3 *In vivostudies*

#### 2.3.1 Drugs and chemicals

Silymarin, isoniazid, rifampicin, pyrazinamide, ethambutol, ketamine hydrochloride, distilled water, surgical spirit, carboxy methyl cellulose, formaldehyde and ethanol.

#### 2.3.2 Animals

Wistar albino rats of both sexes weighing 150–200 g were chosen as subjects for this investigation. The rats were kept in polypropylene cages under controlled conditions, such as a temperature of 24 °C ± 2 °C, a light‒dark cycle of less than 12 h, and *ad libitum* access to a standard pellet diet and water. Rats were subjected to an acclimatization period before the experiment. The Institutional Animal Ethics Committee approved the study procedure, which followed the rules established by the CCSEA [20].

#### 2.3.3 Acute toxicity studies

Acute toxicity studies were performed as per OECD 423 guidelines, and the flavonoid-rich fraction of the extract of *Sesbania grandiflora* (EESG) was administered to albino Wistar rats at concentrations ranging from 5 mg/kg to 2000 mg/kg [20]. The animals were monitored for behavioural changes, signs of toxicity, and any other changes continuously and observed for any lethality for 14 days, after which the desired dose was selected.

#### 2.3.4 Grouping of animals

Group 1: Control (distilled water, p.o. for 28 days)
Group 2: Toxicant group (antitubercular drugs, 0.5 ml, i.p.)
Group 3: Standard drug + Anti-tuberculosis drugs (silymarin 100 mg/kg p.o. for 28 days)
Group 4: Anti-tubercular drugs + *EESG* (250 mg/kg) p.o. for 28 days
Group 5: Anti-tubercular drugs + *EESG* (500 mg/kg) p.o for 28 days

#### 2.3.5 Experimental design

For the investigation, male and female albino rats were divided into five groups of 6 animals each. The rats were given vehicle, *Sesbania grandiflora* fractions at doses of 250 mg/kg and 500 mg/kg, or silymarin at a dose of 100 mg/kg twice daily [21]. This pretreatment was given 1 h before the daily dose of antitubercular medications [22]. Apart from the normal control group, all other groups received a daily dose of antitubercular medications consisting of isoniazid, rifampicin, pyrazinamide, and ethambutol for 28 days [23,24].

#### 2.3.6 Biochemical estimations

The animals were anaesthetized with ketamine hydrochloride on the 28th day of the investigation, and blood samples were taken through heart puncture. Standard techniques were used to measure the levels of total bilirubin (TB), triglycerides, serum glutamic oxaloacetic transaminase (SGOT), serum glutamic pyruvic transaminase (SGPT) [25] and total proteins (TP) [22,26]. Following blood collection, the animals were euthanized, and their livers were immediately separated and stored in the cold. The liver tissue was crosscut into tiny slices with a medical blade and then blotted on filter paper. The tissue was then chopped and homogenized in 10 mM Tris-HCl buffer (pH 7.4) at a 10% w/v concentration using a glass homogenizer with a tight Teflon pestle. The homogeneity was accomplished with 25 strokes at 2500 rpm. The resulting clear supernatant was utilized for assessing the activity of oxidative stress markers, such as Catalase (CAT), Reduced glutathione (GSH), and superoxide dismutase (SOD) [26].

#### 2.3.7 Histopathological studies

A modified Luna approach was used to examine the liver histopathologically. Autopsied livers were cleaned with normal saline before being fixed in 10% formalin for 2 h and then immersed in bovine solution for 6 h [27]. Next, the fixed livers were embedded in paraffin, and microtome sections of 5 μm thickness were made. These sections were treated with alcohol-xylene and stained with hematoxylin. Finally, the liver slices were inspected under a light microscope to determine whether there were any histological abnormalities or damage [22].

### 2.4 *In vitro* cytotoxicity studies

#### 2.4.1 Reagents and chemicals

Dulbecco's Modified Eagle Medium (DMEM)- Low Glucose - (#AL149, Himedia), Fetal Bovine Serum (#RM10432, Himedia), MTT Reagent (5 mg/ml) (# 4060 Himedia), DMSO (#PHR1309, Sigma), l-ornithine-l-aspartate salt (LOLA) (#07125, Sigma), Absolute ethanol (#24102, Sigma), D-PBS (#TL1006, Himedia). Using chemicals and reagents, we performed the experiment on Stellixir Bio Pvt. Ltd. as a platform.

#### 2.4.2 Cell lines and maintenance

HepG2 cells (a human liver cancer cell line) were procured from Stellixir Bio Pvt. Ltd., Bangalore, India. When grown in low-glucose DMEM, the cells were treated with 10% Fetal Bovine Serum (FBS) and 1% antibiotic-antimycotic solution and subcultured every two days in an environment of 5% CO_2_ and 18–20% O_2_ at 37 °C [28].

#### 2.4.3 Estimation of the MTT assay

In a 96-well plate, 200 μL of cell suspension was added at a suitable cell density (20,000 cells per well). The cells were allowed to develop for approximately 24 h before the medium was removed. Next, the test reagent was added at the designated concentrations [29]. The cells were incubated in a 100 mM ethanol solution for 2 h before extraction. Both the plates and the spent medium were removed from the incubator, and MTT reagent was added to achieve a final concentration of 0.5 mg/mL of the total volume after 24 h of incubation at 37 °C in a 5% CO_2_ environment [30]. To prevent light interference, the plate was covered with aluminium foil. The plates were incubated for an additional 3 h in the incubator. The MTT formazan crystals were dissolved by adding a solubilization solution such as DMSO. Gentle agitation, by using some tools such as a gyratory shaker, can aid in the dissolving process [31]. In some cases, particularly with thick cultures, pipetting up and down may be needed to fully dissolve the MTT crystals. The absorbance was measured at 570 nm and 630 nm using a spectrophotometer or an ELISA reader [32,33]. Using a linear regression equation (y = mx + c), the M and C values for y = 50 were determined from the viability graph. The percentage of viable cells was calculated using the following formula:

% Cell viability = (mean absorbance of treated cells/mean absorbance of untreated cells) × 100.

### 2.5 Estimation of antioxidant parameters

Samples of *in vitro* antioxidant parameters were obtained from Stellixir Bio. Pvt. The data were analysed according to standard procedures.

#### 2.5.1 Total glutathione (GSH) assay

In a 96-well plate, 25 μL of 1X glutathione reductase solution was added to each well for EESG extraction. Then, 25 μL of the 1X NADPH solution and 100 μL of the prepared glutathione standards or samples were added to each respective well. Finally, 50 μL of 1X chromogen was added and mixed briefly, the absorbance was recorded at 405 nm at 2 min intervals for 10 min, and the concentrations of both the standards and the samples were calculated [22,34].

#### 2.5.2 Catalase (CAT) assay

The standards were diluted to 100 mL (90 mL of standard + 10 mL of stock solution). The volume of the positive control was adjusted to 78 mL with CAT assay buffer. After the addition of the reagent solution, the volume was adjusted to 78 mL with CAT assay buffer. Then, 10 mL of Stop Solution was added to each High control (HC) sample well for CAT inhibition [35]. To completely suppress CAT activity in the HC sample wells, the samples were incubated at 25 °C for 5 min [22]. The CAT reaction was started by adding 12 mL of fresh 1 mM H_2_O_2_ to each of the sample, positive control, and sample HC wells. The cells were incubated for 30 min at 25 °C. To prepare 50 mL for each reaction, 10 mL of developer was added. Fifty millilitres of Developer Mix were added to each standard, sample, sample HC, and positive control well. The mixture was incubated for 10 min at 25 °C in the dark [36].

#### 2.5.3 Superoxide dismutase (SOD) assay

The components, except lysates, were maintained at room temperature. The total reaction volume was 1.5 mL, and the reagent volume was 107.5 μL. A volume of 107.5 μL was subtracted, and the sample volume was 1500 μL to determine the water volume. Briefly, each reagent was vortexed before use. In a cuvette, 25X reaction buffer (60 μL), xanthine solution (7.5 μL), and water were added (R. Harish, T. Shivanandappa, 2004) to reach a volume of 1500 μL. The solution was thoroughly mixed before use, 30 μL of Nitro blue Tetrazolium Chloride (NBT) solution was added, and the solution was mixed vigorously. The lysate was added and vortexed for 5 min. Briefly, the XOD solution was vortexed, 10 μL was added to the cuvette, and the mixture was vortexed for 5 min. After 5–6 min, the absorbance was recorded at 450 nm using an ELISA reader [37].

### 2.6 Molecular docking method

A comprehensive literature search identified fifteen flavonoid-flavanol chemical compounds abundantly present in the plant leaf extract. These compounds include baicalein, biochanin A, chrysin, daidzein, eriodictyol, formononetin, genistein, glycitein, hesperetin, isorhamnetin, kaempferol, luteolin, myricetin and naringenin. Human peroxisome proliferator-activated receptors are ligand-dependent transcription factors and are among the most significant therapeutic targets for liver disease. The Protein Data Bank (PDB ID: 3VI8) provided the crystal structure of the target peroxisome proliferator-activated receptor alpha (PPARα) [38]. The three-dimensional structures of the fifteen flavonoid phytochemicals used for the docking study were retrieved from the PubChem database (https://pubch em. ncbi. nlm. nih.gov/) in the SMILES format, which was then translated to the corresponding MOL2 forms using CHEMDRAW 11 software. Using the docking module built into Molecular Operating Environment software version (MOE 2015.1001) [39], docking simulations were run with 15 flavonoid phytochemicals in the ligand binding domain of PPARα to examine potential binding interactions and mechanistic inhibitors (MOE, 2015). The initial low-energy conformations of the flavonoid-flavanol molecules were generated using the low-molecular-weight MD method with the MMFF94x force field and Gasteiger-Huckel charges. This process involved 10,000 iterations with an RMS gradient of 0.01 kcal. To address protein structural issues, polar hydrogen atoms were added, and the hydrogen bond network was optimized. The MMFF94x force field configuration and partial charges were applied to the protein. The inhibitor-binding pocket was identified using the site finder module in the MOE program. The analysed binding pocket of PDB: 3VI8 contains several key amino acid residues. These include TYR214, PHE218, ASN219, MET220, ASN221, LYS222, ILE241, LEU247, ALA250, GLU251, LEU254, VAL255, LEU258, ARG271, ILE272, PHE273, HIS274, CYS275, CYS276, GLN277, CYS278, THR279, SER280, GLU282, THR283, THR285, GLU286, TYR314, ILE317, PHE318, MET320, LEU321, SER323, VAL324, MET325, MET330, LEU331, VAL332, ALA333, TYR334, GLY335, ILE339, LEU344, ILE354, MET355, LYS358, ILE375, HIS440, VAL444, LEU456, LEU460, and TYR464. In Fig. 4, the identified binding pocket is represented by the molecular surface contour in green. The ligand was docked into the protein's active site using default parameters, including Placement: triangle matcher, Refinement: rigid receptor, Scoring: London dG and GBVI/WSA dG, with a maximum of 10 conformations. The final output of the docked postures was determined by the final score S, specifically the GBVI/WSA dG score [44]. The optimal flavonoid flavonols were determined by evaluating their binding energy and interactions with amino acid residues in the PPARα protein.

### 2.7 Statistical analysis

Statistical analysis was conducted using GraphPad Prism software, and further analysis was refined using Cell Quest Software, version 6.0. The data are presented as the mean ± SEM, and significant differences were assessed using one-way analysis of variance followed by Dunnett's T test. A p value less than 0.005 was considered to indicate statistical significance.

## 3 Results

### 3.1 Extraction and fractionation

The crude extract of whole parts of *Sesbania grandiflora* was fractionated [19] to yield flavonoid-rich fractions, and the percentage yield was found to be 6.32% (w/w).

### 3.2 Screening of phytochemical constituents

Phytochemical screening of the crude extract of *Sesbania grandiflora* revealed the presence of flavonoids as major chemical constituents by screening using standard chemical tests such as the Shinoda test; these active principles were responsible for different therapeutic effects.

### 3.3 Acute toxicity studies

According to OECD 423 guidelines, up to 2000 mg/kg/flavonoid-rich fraction of extract of *Sesbania grandiflora* (EESG) did not cause any lethal effects. No signs of changes in the dermatological system, behavioural patterns, salivary secretions or remaining parameters, such as tremors and sleep, were observed in the control rats. Therefore, the desired doses determined as the 1/4th and 1/8th percentiles of 2000 mg/kg/b.w were selected for this study. Hence, *in vivo* experiments were carried out with only one dose (250 mg/kg or 500 mg/kg).

### 3.4 *In vivoresults*

#### 3.4.1 Hepatoprotective studies

The administration of antitubercular drugs led to a notable increase in serum enzymes such as SGPT and SGOT, as well as elevated levels of triglycerides and total bilirubin, as shown in Table 1. Conversely, total protein levels were decreased compared to those in the normal control group. Assessment of *in vivo* antioxidant parameters, including catalase, lipid peroxidation, and SOD, revealed a decrease in these parameters compared to those of the normal control group, as shown in Table 1. However, pretreatment with silymarin and SG extract significantly mitigated the biochemical changes induced by the antitubercular drugs.

**Table 1:** Effects of *Sesbania grandiflora* on liver and antioxidant markers.

Liver and antioxidant markers	Control group	Negative control group	Test groups		
			Silymarin + Anti TB drugs	EESG 250 mg + Anti TB drugs	EESG 500 mg + Anti TB drugs
**Liver markers**					
SGOT(IU/L)	30 ± 0.16	124.5 ± 5.38	51.5 ± 0.62**	90.6 ± 0.92*	72.5 ± 0.61**
SGPTs (IU/L)	34.2 ± 0.21	175.5 ± 3.12	83.8 ± 0.67**	122.3 ± 4.62*	111.5 ± 5.26*
TGs(mg/dL)	83.8 ± 0.52	195.5 ± 4.68	133 ± 6.41**	162.6 ± 3.62*	145.2 ± 2.08**
Total bilirubin (IU/L)	0.205 ± 0.02	1.258 ± 0.06	0.356 ± 0.07**	0.562 ± 1.02*	0.403 ± 0.06**
Total protein(g/dL)	5.5 ± 0.92	3.1 ± 0.08	5.15 ± 1.2**	3.4 ± 0.82*	5.5 ± 0.04*
**Antioxidant markers**					
Catalase(μmol/mg)	88.33 ± 6.02	31.18 ± 3.64	75.85 ± 4.27**	41.095 ± 1.23*	60.825 ± 2.44*
Superoxide dismutase (SOD) (unit/mg)	13.1 ± 0.86	4.17 ± 0.04	8.78 ± 0.08**	6.15 ± 1.04*	7.91 ± 0.14**
Lipid peroxidation(μg/mg)	5.83 ± 0.19	8.16 ± 0.17	6.11 ± 0.09**	7.44 ± 0.32*	5.06 ± 0.46*

Notes: Values are expressed as Mean ± SEM of 6 rats in each group. *P < 0.05, **P < 0.01 compared to negative control Group.

#### 3.4.2 Histopathological results

**Fig. 1:**
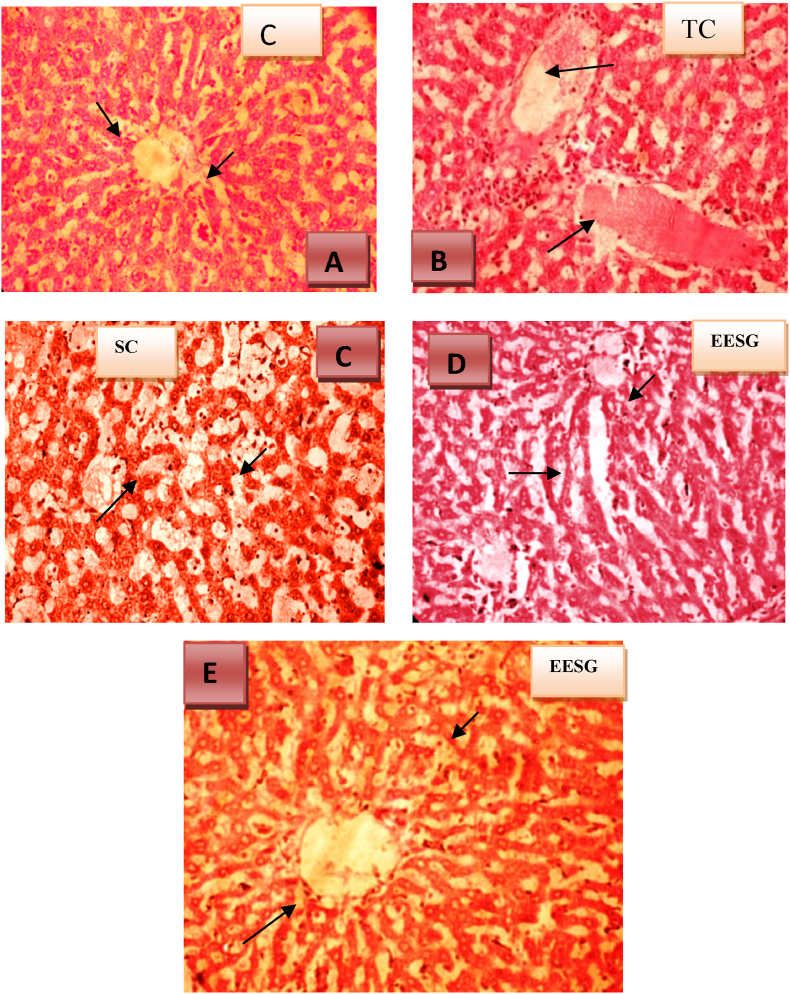
Histopathological analysis of liver tissue. **C:** In the standard silymarin-treated group (100 mg/kg), sinusoidal congestion and periportal mononuclear inflammatory infiltration were observed. **E:** In the *Sesbania grandiflora*-treated group (500 mg/kg), the liver exhibited an intact architecture, a few regenerative hepatocytes, and sinusoidal congestion. Most sinusoids and central veins appear dilated and congested (short arrow). Scattered regenerative hepatocytes (long arrow) are observed. There was sparse, scattered mononuclear inflammatory infiltration among hepatocytes. **D:** In the *Sesbania grandiflora*-treated group (250 mg/kg), the liver exhibited an intact architecture, apoptotic and regenerative hepatocytes, sinusoidal congestion, and aggregates of histiocytes. Some sinusoids show congestion. Scattered apoptotic hepatocytes (short arrow) and regenerative hepatocytes (long arrow) are observed. Intervening the hepatocytes, there are aggregates of histiocytes (med arrow) and scattered mononuclear inflammatory cells. **B:** In the presence of the antitubercular drug toxicant, the liver shows a partially effaced architecture, degenerative changes in hepatocytes, the presence of epithelioid granulomas, and aggregates of mononuclear inflammatory cells. Some sinusoids exhibit congestion (short arrow), and most hepatocytes display degenerative changes (long arrow). Epithelioid granulomas and aggregates of mononuclear inflammatory cells are observed within the parenchyma. **A:** The studied section of the liver exhibits preserved architecture, with most of the perivenular (zone-3), periportal (zone-1), and mid-zonal (zone-2) hepatocytes appearing normal. Scattered mononuclear inflammatory cells are observed within the hepatic parenchyma.

In the normal control group, hepatocytes exhibited a normal lobular architecture in the liver. However, in the group treated with antitubercular drugs, the liver displayed microvascular fatty changes, partially distorted architecture, degenerative changes in some hepatocytes, the presence of epithelioid granulomas, and clusters of mononuclear inflammatory cells. Conversely, in the groups pretreated with silymarin and SG leaf extract, minimal fatty acid changes were observed, and their lobular architecture appeared normal. These findings indicate that SG leaf extract possesses significant hepatoprotective activity, as depicted in Fig. 1.

### 3.5 Cytotoxicity results

#### 3.5.1 MTT assay

The MTT assay results suggested that the test compound extract showed moderate cytotoxicity against human liver cancer cells at a concentration of 200 μg/mL with an IC_50_ value of 190.28 μg/ml, and the remaining concentrations were nontoxic. Based on the preliminary MTT assay results, we determined the optimum extract concentration at concentrations of 50 μg/ml and 100 μg/ml, as indicated in Table 2. Furthermore, HepG2 cells pretreated with 100 mM ethanol in combination with nontoxic concentrations of 50 μg/ml and 100 μg/ml LOLA and the hepatoprotective agent LOLA at concentrations of 0.01 μg/ml and 0.01 μg/ml to induce hepatotoxicity. The results confirmed the dose-dependent hepatoprotective potency of the extract in ethanol-induced cells and showed similar results to those of LOLA, a commercially available hepatoprotective drug, by increasing the percentage of viable cells, as depicted in Table 2.

**Table 2:** Viability of HepG2 cells treated with test compound and control.

S.No	Different concentrations of SG extract and LOLA conjugated with ethanol and control	% viability of HepG2 cells
1.	Untreated	100
2.	Ethanol-100 mM	18.17
3.	12.5 μg/ml	99.09
4.	25 μg/ml	97.03
5.	50 μg/ml	88.85
6.	100 μg/ml	74.19
7.	200 μg/ml	47.54
8.	Extract-50 μg	90.82
9.	Extract-100 μg	75.4
10.	Ethanol 100 mM + Extract-50 μg	58.78
11.	Ethanol 100 mM + Extract-100 μg	67.36
12.	Ethanol 100 mM + LOLA 0.01 μg/ml	52.76
13.	Ethanol 100 mM + LOLA 0.1 μg/ml	70.45

#### 3.5.2 Antioxidant parameters

In this study, extracts at different concentrations were combined with LOLA and ethanol at various concentrations and evaluated by ELISA to measure the CAT, SOD and GSH levels in HepG2 pelleted cells. Increased levels of free radical molecules cause oxidative stress in cells, which results in damage to macromolecules such as DNA, proteins, and lipids. The concentrations of the compounds used to treat the cells are listed in Table 3 (CAT concentrations), Table 4 (SOD concentrations) and Table 5 (GSH concentrations). Catalase is an antioxidant enzyme that is widely distributed in various organisms. Its primary function is to facilitate the decomposition of hydrogen peroxide (H_2_O_2_) into water and oxygen. Superoxide dismutase (SOD), on the other hand, plays a crucial role in neutralizing superoxide radicals (O_2_-) by converting them into hydrogen peroxide (H_2_O_2_) and elemental oxygen (O_2_), thereby protecting against the harmful effects of superoxide radicals. Within cells, glutathione exists in two forms: reduced (GSH) and oxidized (GSSG). Glutathione is actively involved in the breakdown of peroxides and plays a regulatory role in the nitric acid cycle.

**Table 3:** Catalase activity observed in experimental groups.

Culture condition	CAT activity (mU/mL)
Untreated	7.65 ± 0.021
Ethanol-100 mM	0.66 ± 0.043
Extract-100 μg	7.44 ± 0.027
LOLA-0.1 μg	7.51 ± 0.032
Ethanol + Extract-100 μg	3.34 ± 0.034*
LOLA + Extract-100 μg	7.56 ± 0.029**

The values are expressed as the means ± SEMs; ethanol-treated group vs untreated group; *p < 0.05, **p < 0.01.

**Table 4:** Concentrations of SOD in experimental groups.

Culture condition	SOD Concentration (Unit/Min/mg)
Untreated	5.19 ± 0.121
Ethanol-100 mM	0.99 ± 0.063
Extract-100 μG	5.25 ± 0.028
LOLA-0.1 μG/ml	5.21 ± 0.021
Ethanol + Extract-100 μG	3.29 ± 0.087*
LOLA + Extract-100 μG	5.19 ± 0.075*

The values are expressed as the means ± SEMs; ethanol-treated group vs untreated group; *p < 0.05, **p < 0.01.

**Table 5:** GSH concentrations in experimental groups.

Sample	GSH concentration (μM)
Untreated	0.476 ± 0.069
Ethanol-100 mM	0.056 ± 0.047
Extract-100 μG	0.474 ± 0.056
LOLA-0.1 μG/ml	0.483 ± 0.087
Ethanol + Extract-100 μG	0.351 ± 0.053*
LOLA + Extract-100 μG	0.482 ± 0.021*

The values are expressed as the means ± SEMs; ethanol-treated group vs untreated group; *p < 0.05, **p < 0.01.

**Fig. 2:**
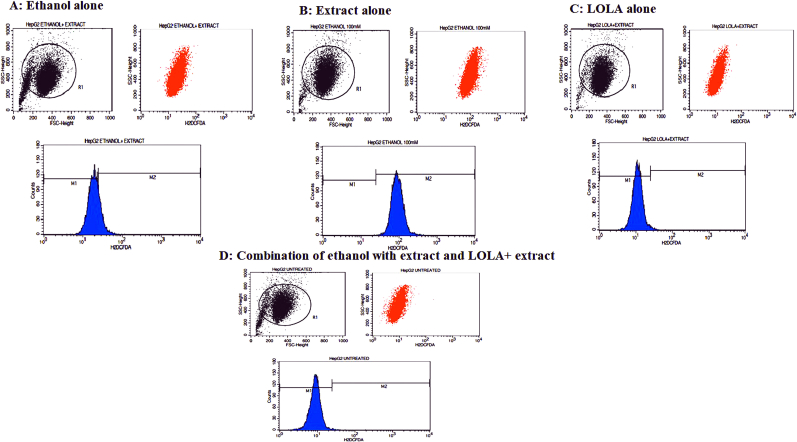
Histogram studies of HepG2 cell lines. A: Ethanol alone, B: extract alone, C: LOLA alone and D: combination of ethanol with extract and LOLA + extract-treated HepG2 pelleted cells.

Scavenging measurements are the extent of the antioxidant capacity of the given samples. After supplementation, the amount of glutathione (GSH) increased to 0.351 in the ethanol+100 μg extract group and 0.482 in the LOLA+100 μg extract group, and the activity of antioxidants such as catalase (CAT) increased to 3.34 in the ethanol+100 μg extract group and 7.56 in the LOLA+100 μg extract group. SOD activity also increased in the ethanol+100 μg extract group at 3.29 μg and in the LOLA+100 μg extract group at 5.19 μg. The possibility of the flavonoid-rich fraction of *Sesbania grandiflora* plant extracts generating free radicals was evaluated using the 2′,7′-dichlorodihydrofluorescein diacetate (H2DCFDA) assay (Fig. 2).

#### 3.5.3 DCF mean fluorescence intensity

**Fig. 3:**
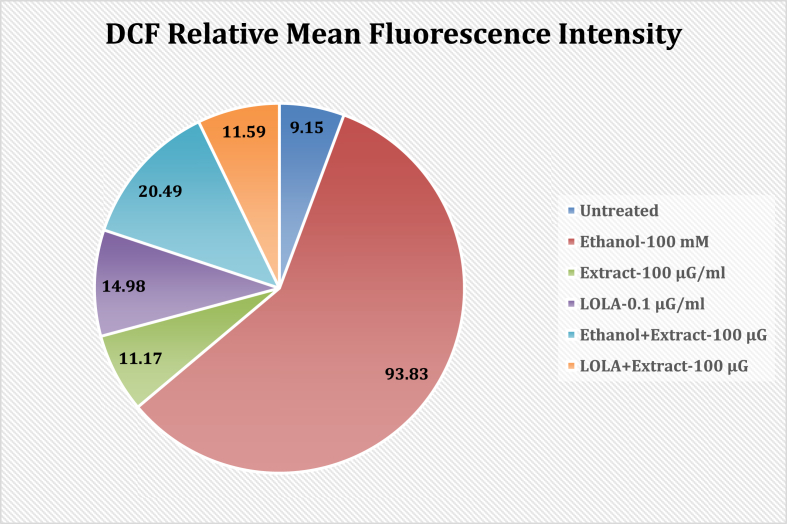
Different culture conditions showing the relative mean fluorescence intensity of DCF in HepG2 cells treated with Untreated, Ethanol alone, Extract alone, LOLA alone and the combination of Ethanol with Extract and LOLA with Extract.

The plant extract significantly reduced the mean Dichlorofluorescein (DCF) fluorescence intensity in an ethanol-stimulated paradigm, confirming its hepatoprotective properties. Untreated cells exhibited DCF intensity with a relative mean fluorescence intensity of 9.15, while ethanol DCF expression was 93.83 and 20.49 for the alone and ethanol-conjugated extracts, respectively. The combination of LOLA and Extract yielded comparable findings, with 11.59 DCF expressing. Overall, our observations indicate that the extract greatly decreased the DCF concentration. Its expression in ethanol-induced liver cells (HepG2) showed strong hepatoprotective potential, as shown in Fig. 3.

#### 3.5.4 Analysis of the binding site interactions of PPARα proteins with flavonoid flavonols

**Fig. 4:**
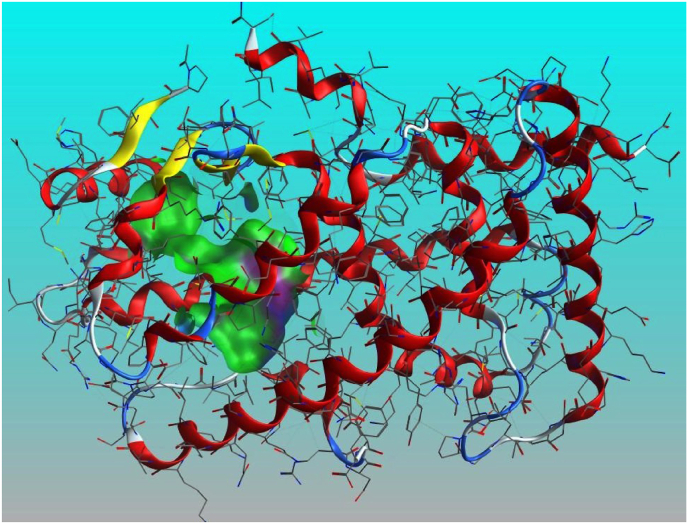
The binding site in the peroxisome proliferator-activated receptor alpha protein is shown by the green surface highlight.

**Fig. 5:**
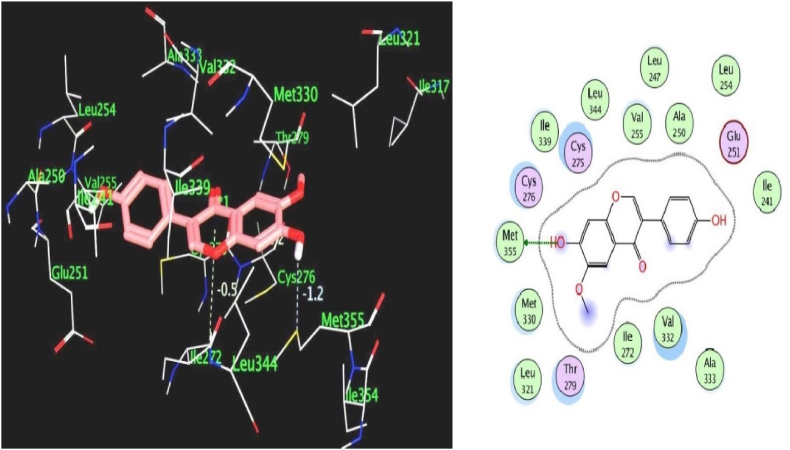
The 3D & 2D representation of the binding pattern and interaction of glycitein molecules in the active site of the PPARα protein.

**Fig. 6:**
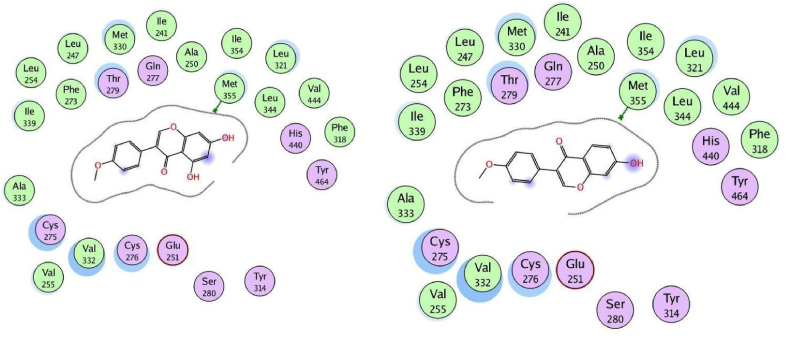
Two-dimensional illustration of docked biochanin A (left) and formononetin (right) at the active site of the PPARα protein.

**Fig. 7:**
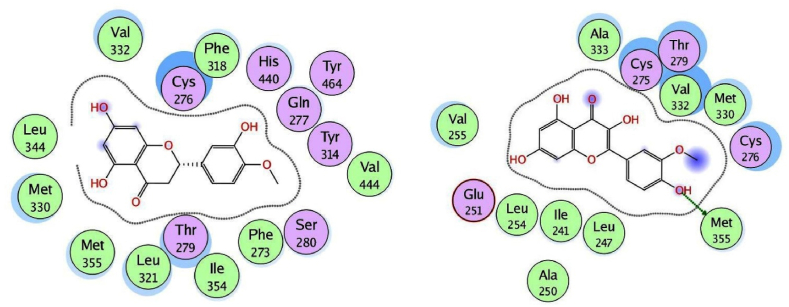
Two-dimensional illustration of docked hesperetin (left) and isorhamnetin (right) at the active site of the PPARα protein.

**Fig. 8:**
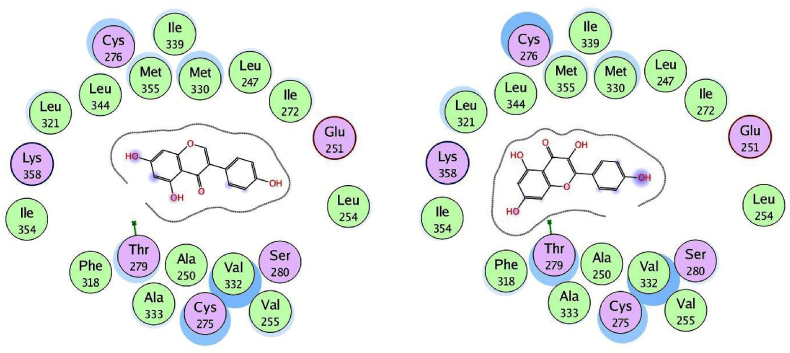
Two-dimensional illustration of docked genistein (left) and kaempferol (right) at the active site of the PPARα protein.

**Fig. 9:**
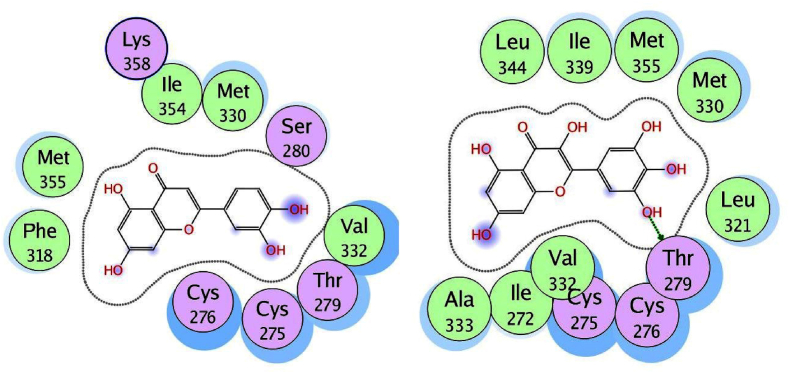
Two-dimensional illustration of docked luteolin (left) and myricetin (right) at the active site of the PPARα protein.

We used the MOE program to perform molecular docking investigations on all fifteen flavonoid-flavanol compounds to obtain more precise information about the interactions between the selected flavonoid flavonols and PPARα and to determine their important hepatoprotective effects. All fifteen flavonoid flavonols (kcal/mol ranging from −6.34 to −7.35) markedly inhibited the target PPARα proteins (Table 4). The phytochemicals most prone to bind to the PPARα protein were biochanin A (−6.99 kcal/mol), glycitein (−7.35 kcal/mol), formononetin (−6.92 kcal/mol), hesperetin (−7.00 kcal/mol), and isorhamnetin (−7.16 kcal/mol). All the compounds with the highest binding affinities were predicted to have methoxy substituents connected to the phenyl and chromen-4-one rings, in contrast to the compounds with moderate binding affinities (<−6.99 kcal/mol), which only have hydroxyl substituents on the aromatic rings. Among the strong binding site affinities of flavonoid-flavanol compounds (Table 6), glycitein had the highest binding affinity for PPARα (7.35 kcal/mol). The ligand is securely positioned by the active site, taking up the entire pocket of the protein (Fig. 4). This glycitein compound's binding mechanism consists primarily of hydrophobic interactions, with minor polar and hydrogen bond interactions. The polar hydroxyl groups and oxygen atoms of the chromen-4-one rings interact with ions from the acidic regions of CYS276 and CYS275. The hydroxyl group of the molecule and the sulfur atom of the side chain SCH3 group of MET355 are connected by hydrogen bonding because the MET355 residue is extremely close to the hydroxyl of the chromen-4-one ring. Remarkably, CH-π interactions are used by the chromen-4-one ring to engage with the methyl group of the ILE272 amino acid. Acidic polar contacts arise from glycitein's phenolic hydroxyl groups projecting towards the carboxylate groups of the GLU251 residue. Strong hydrophobic interactions exist between the larger amino acid residues surrounding the phenyl and chromen-4-one rings (Fig. 5). These residues are LEU344, ILE339, VAL 255, LEU247, ALA250, LEU254, ILE241, VAL 332, ALA333, MET330, and LEU32. Promising high-binding candidates for targeting PPARα proteins, such as biochanin A, hesperetin, formononetin, and isorhamnetin, were also identified by docking analysis. By π-π stacking and alkyl and π-alkyl interactions with the Ala250, Phe273, Leu247, Val255, Val332, Met330, Ile241, Phe318, Ile339, Met355, Leu344, and Val444 residues, the two benzene rings of biochanin A and formononetin compounds are related to hydrophobic interactions. With the residues Cys275, Glu251, Gln277, Cys276, Thr279, Tyr464, and His440, the hydroxyl and/or carbonyl groups formed typical hydrogen bonds. Fig. 6 shows that the active region of the PPARα protein interacts with the flavonoids biochanin A (left) and formononetin (right). With respect to the PPARα proteins, the aromatic groups of hesperetin formed alkyl and π-alkyl linkages with the residues Val332, Phe318, Leu344, Met330, Met355, Leu321, Ile354, Phe373, and Val44. The polar groups of the hesperetin complex interact with the residues Cys276, His440, Gln277, Tyr464, Tyr314, Thr279, and Ser280. The isorhamnetin compound had comparable hydrophobic and lowest polar contacts by networking with the residues Val255, Leu254, Ile241, Leu247, Ala250, Ala333, Val332, Met330, Gln251, Cys275, Thr279, Cys276 and Met355. The interactions between the active region of the PPARα protein and the flavonoids hesperetin (left) and isorhamnetin (right) are shown in Fig. 7. Moreover, we investigated the binding patterns of a few moderate binding affinity chemicals, such as genistein (−6.83 kcal/mol), myricetin (−6.50 kcal/mol), kaempferol (−6.50 kcal/mol), and luteolin (−6.77 kcal/mol). The amino acid residues Leu321, Leu344, Met355, Ile339, Met330, Leu247, Ile272, Leu254, Ile354, Phe318, Ala333, Ala250, Val352, and Val255 participate in hydrophobic interactions with the 2-phenyl-1,4-benzopyrone moiety of genistein and kaempferol. The hydroxy groups of these compounds function through networks of hydrogen bonds with the Lys358, Thr279, Ser280, Cys275, and Glu251 residues (Fig. 8). Hydrophobic and polar interactions with the residues Phe318 and Met355 stabilize the molecules luteolin and myricetin. Hydrophobic and polar contact residues (Ala333, Cys276, Cys275, Thr279, Leu321, Ile354, Met330, Val322, Lys358, and Ser280) are distributed equally in both compounds because of polyphenolic groups encircling the core of the 2-phenyl-1,4-benzopyrone moiety (Fig. 9). Therefore, it seems that flavonoid-flavanol phytochemicals may have additional advantages in terms of altering their hepatoprotective effects since they can interact with PPARα. Moreover, the interactions of these molecules offer new directions for the study and management of diseases resulting from liver injury, as well as benefits over currently approved FDA drugs.

**Table 6:** Docking studies and binding energy of phytochemical flavonoid flavonols against the PPARα target protein [[40], [41], [42], [43]].

S.No	Flavonoids-flavanols	PubChem CID	Structure	S score (kcal/mol)
1	Baicalein	5281605		−6.34
2	Biochanin A	5280373		−6.99
3	Chrysin	5281607		−6.47
4	Daidzein	5281708		−6.64
5	Eriodictyol	440735		−6.70
6	Formononetin	5280378		−6.92
7	Genistein	5280961		−6.83
8	Glycitein	5317750		−7.35
9	Hesperetin	72281		−7.00
10	Isorhamnetin	5281654		−7.16
11	Kaempferol	5280863		−6.50
12	Luteolin	5280445		−6.77
13	Myricetin	5281672		−6.50
14	Naringenin	932		−6.48
15	Quercetin	5280343		−6.42

## 4 Discussion

The liver, a vital organ in the human body, is susceptible to injury caused by various chemicals and drugs. In this study, we used LOLA as a combination drug to protect against liver damage due to its clinical relevance. Chronic alcoholic patients often exhibit hepatomegaly, characterized by the accumulation of lipids and proteins in hepatocytes and impaired protein secretion. During hepatic damage, cellular enzymes such as serum transaminases are released from liver cells into the bloodstream, leading to increased concentrations [45]. In our study, we administered antitubercular drugs (a combination of isoniazid, rifampicin, pyrazinamide, and ethambutol) as toxicants to induce liver damage, considering their clinical significance. These drugs are known to induce various detrimental effects on the liver in a dose-dependent manner.

Chronic alcohol consumption and 28 days of antitubercular drug administration resulted in increased serum SGPT and SGOT enzyme levels [46]. However, the administration of the ethanolic extract of *Sesbania grandiflora* significantly reduced the serum transaminase enzyme levels and increased the total protein and albumin levels. These findings indicate the hepatoprotective effect of *Sesbania grandiflora* extract against liver cell damage induced by antitubercular drugs [47].

A considerable decrease in the levels of SGOT, SGPT, bilirubin, and triglycerides in the treated group, together with an increase in total proteins, indicated a hepatoprotective effect. Lower SGOT and SGPT levels imply less liver inflammation or cellular damage. Reduced bilirubin levels indicate better liver function and less bile blockage [48]. Triglyceride reduction indicates improved lipid metabolism and a decreased risk of fatty liver disease. Finally, a rise in total proteins suggests increased protein synthesis and liver function.

The hepatoprotective effects of *Sesbania grandiflora* extract were further confirmed through histopathological observations. Overdoses of antitubercular drugs are known to cause partially affected architecture, degenerative changes in hepatocytes, epithelioid granulomas, and aggregates of mononuclear inflammatory cells in the liver [49]. Fatty liver, characterized by macrovascular steatosis and ballooning of hepatic cells, is also observed. Alcoholic fatty liver is influenced by increased substrate supply for fatty acid esterification, stimulation of the esterification pathway, and decreased export of triglycerides such as very-low-density lipoproteins (VLDLs) from the liver [50].

Pretreatment with the ethanolic extract of *Sesbania grandiflora* significantly mitigated these effects. Microvascular inflammation induced by antitubercular drugs was markedly reduced following extract pretreatment. Consequently, the accelerated recovery of hepatic cells, as evidenced by histopathological observations, suggests that *Sesbania grandiflora* extract protects against membrane fragility, thus reducing the leakage of marker enzymes into the bloodstream [51].

Fractionation of *Sesbania grandiflora* revealed the presence of a rich content of flavonoids that possess potential hepatoprotective activity and cytoproctective activities. Serum transaminases, which are found in liver cells, enter the serum after hepatic injury, resulting in increased quantities of transaminases. The MTT assay results suggested that the test compound extract showed moderate cytotoxicity against human liver cancer cells at a concentration of 200 μg/ml with an IC_50_ value of 190.28 μg/ml, and the remaining concentrations were nontoxic in nature [25]. Based on the preliminary MTT assay results, we decided on the optimum concentration of extract at 50 μg/ml and 100 μg/ml and further performed combination treatment with nontoxic concentrations of 50 μg/ml and 100 μg/ml and the hepatoprotective agent LOLA at concentrations of 0.01 μg/ml and 0.01 μg/ml on HepG2 cells that were prestimulated with 100 mM ethanol to induce hepatotoxicity. The results confirmed the dose-dependent hepatoprotective potency of the extract in ethanol-induced cells and showed similar results to those of LOLA, a commercially available hepatoprotective drug, by increasing the percentage of viable cells.

To maintain cellular functionality, cells need to possess the ability to combat the damaging effects of free radicals. This is achieved through endogenous systems comprising various enzymes, including superoxide dismutase (SOD), catalase (CAT), and glutathione (GSH). The considerable antioxidant capacity exhibited by *Sesbania grandiflora* extracts suggests their potential therapeutic utility in diseases associated with excessive production of reactive oxygen species [51,52]. Given its wide availability, *Sesbania grandiflora* may be beneficial for the prevention of various diseases. After treatment with ethanol plus extracts, LOLA, and the combination of extracts, the levels of antioxidant parameters such as GSH, CAT, and SOD exhibited slight increases, with the latter showing statistically significant improvements.

The extract demonstrated significant hepatoprotective effects by markedly reducing the mean DCF fluorescence intensity in an ethanol-stimulated model. Untreated cells displayed a relative mean fluorescence intensity of 9.15, while those exposed to ethanol alone showed a DCF expression of 93.83. However, cells treated with the extract in combination with ethanol exhibited markedly reduced DCF expression. Similar results were observed with the combination of LOLA and the extract, showing a DCF expression of 11.59. Overall, these findings strongly suggest that the extract effectively mitigated DCF expression in ethanol-induced liver cells (HepG2), indicating promising hepatoprotective potential.

Molecular docking analyses of fifteen flavonoid-flavanol compounds were conducted using MOE software to determine their interactions with PPARα proteins. The phytochemicals biochanin A, glycitein, formononetin, hesperetin, and isorhamnetin were found to most likely bind to the PPARα protein. Among the flavonoid-flavanol compounds with the highest binding site affinities, glycitein had the highest binding affinity for PPARα, with a value of 7.35 kcal/mol. The binding mechanism consists primarily of hydrophobic interactions, with minor polar and hydrogen bond interactions. Biochanin A, hesperetin, formononetin, and isorhamnetin were identified as potential high-binding phytochemicals for targeting PPARα proteins. This study also investigated the binding interaction patterns of moderate binding affinity compounds, including genistein, kaempferol, luteolin, and myricetin. The 2-phenyl-1,4-benzopyrone moieties of genistein and kaempferol have hydrophobic interactions with amino acid residues, while luteolin and myricetin are stabilized via hydrophobic and polar interactions. The interaction patterns of these molecules provide fresh leads for the development and treatment of liver damage illnesses and offer advantages over existing FDA medications. These PPAR interactions highlight the potential of flavonoid-flavanol phytochemicals for hepatoprotective regulation and provide promising possibilities for liver injury treatment because of their potential hepatocurative properties.

## 5 Conclusion

The findings of this study provide evidence that the flavonoid chemical constituents present in the ethanolic extract of *Sesbania grandiflora* play a crucial role in the treatment of hepatotoxicity and cytotoxicity. An *in vivo* study demonstrated that the constituents of *Sesbania grandiflora* significantly enhance the body's natural antioxidant defense, leading to a reduction in oxidative stress caused by antitubercular drugs, increased levels of CAT, SOD, and GSH antioxidants and a decrease in elevated liver enzyme levels. The results emphasize the hepatoprotective activity of *Sesbania grandiflora* and suggest that this activity can be attributed to its active principles, including flavonoid compounds. These findings present opportunities for further research on the development of potent phytomedicines derived from *Sesbania grandiflora* for hepatoprotection.

The *in vitro* study demonstrated that the flavonoid-rich fraction from *Sesbania grandiflora* significantly suppressed the mean fluorescence intensity of DCF in an ethanol-stimulated model, confirming the cytoprotective potential of SG. Untreated cells exhibited DCF intensity with a relative mean fluorescence intensity of 9.15, while ethanol alone and the ethanol-conjugated extract showed DCF expression levels of 93.83 and 20.49, respectively. The combination of LOLA and the extract also demonstrated similar results, with an 11.59 DCF expression. Overall, these observations strongly suggested that the extract effectively suppressed DCF expression in ethanol-induced liver cells (HepG2) and confirmed its significant cytoprotective potential in HepG2 cell lines, which was supported by molecular docking studies. However, further studies utilizing nontoxic concentrations of the extract at 50 μg/ml and 100 μg/ml are needed to elucidate the molecular mechanisms underlying the hepatoprotective effects of the extract on the HepG2 cell line. Future investigations should also focus on identifying novel molecular mechanisms of the active components derived from *Sesbania grandiflora* that possess hepatoprotective and cytoprotective potential.

## Ethics approval and consent to participate

SVCP/IAEC/I-05/2018-19 dated on 18-11-2019.

## Plant authenticate and specimen voucher number

384, KL-20.

## Source funding

This research did not receive any specific grant from funding agencies in the public, commercial, or not-for-profit sectors.

## Author Contributions

KA, CS, VM: Conceptualization, Methodology, KA, CS: Investigation, Project administration Writing – original draft.

VM, SB, CR, BN: Supervision, Writing– review & editing.

## Declaration of Generative AI in scientific writing

Authors declare that no AI tools were used for writing of the manuscript.

## Declaration of competing interest

All authors declare no conflicts of interest.
